# Locus of control and conformity among adolescents: the mediating role of assertiveness

**DOI:** 10.3389/fpsyg.2026.1704912

**Published:** 2026-04-02

**Authors:** Bereket Merkine Gebresilase, Chuanxia Zhang, Zebdewos Zekarias Elka, Yohannes Bisa Biramo, Esayas Teshome Taddese, Ke Han

**Affiliations:** 1 College of General Education, Shandong Xiehe University, Jinan, China; 2 Department of Psychology, Wolaita Sodo University, Sodo, Ethiopia; 3 Faculty of Education and Liberal Arts, INTI International University, Nilai, Negeri Sembilan, Malaysia

**Keywords:** adolescents, assertiveness, conformity, locus of control, psychological well-being, risky behaviors

## Abstract

**Background/Introduction:**

Adolescents in emerging economies like Ethiopia face unique pressures to conform, often leading to risky behaviors such as substance use. While psychological resources are known to be protective, the pathways through which they influence conformity are not fully understood. This study examined the role of locus of control in predicting adolescent conformity and investigated the mediating effect of assertiveness on this relationship.

**Methods:**

A cross-sectional study was conducted with 516 secondary school students in grades 9–12 (Mage = 14.77 ± 0.71; 52.52% male, 47.48% female). Participants completed validated measures of locus of control, assertiveness, and conformity. Structural equation modeling was employed to test the direct and indirect effects of locus of control on conformity via assertiveness.

**Results:**

Internal locus of control was negatively associated with conformity, while external locus of control showed a significant positive association. Assertiveness also demonstrated a significant negative correlation with conformity. Critically, structural equation modeling revealed that assertiveness partially mediated the relationship between both internal and external locus of control and conformity.

**Conclusion:**

These findings highlight the importance of fostering adolescents’ sense of personal control and assertiveness as protective factors. Interventions that strengthen these psychological resources may help adolescents in high-pressure environments resist peer pressure and make independent, adaptive decisions.

## Introduction

Conformity, broadly defined as the tendency to align one’s attitudes, beliefs, or behaviors with those of a group, is a critical developmental and social phenomenon during late adolescence and young adulthood (Asch, 1956; Nail et al., 2000; Minich et al., 2023). Adolescents, particularly in emerging economies such as Ethiopia, experience significant academic, social, and cultural pressures to conform in order to gain acceptance, avoid rejection, and maintain a sense of belonging. While conformity can serve adaptive purposes by promoting group cohesion and facilitating cultural integration, excessive conformity may undermine individuality, critical thinking, and self-regulation (Hornsey and Jetten, 2004; Cialdini and Goldstein, 2004; Xiao et al., 2025).

Norms implicit rules shared by a group form the foundation of conformity. Individuals often find it socially easier to follow established expectations rather than deviate from them. As a result, conformity may occur both in direct social interactions and in solitary contexts where internalized expectations guide behavior. For instance, individuals may follow social eating habits or media consumption patterns even when alone. However, conformity under peer or group pressure has been linked to risky behaviors including substance use, truancy, delinquency, and early sexual engagement (Namuwonge et al., 2024; Dai et al., 2024; Gazi et al., 2025). Understanding the factors that shape conformity among adolescents and young adults is therefore an important research priority.

A key psychological variable influencing conformity is locus of control (LoC), which reflects the extent to which individuals perceive outcomes as contingent on their own actions versus external forces (Rotter, 1966). Individuals with an internal LoC believe that effort and personal decisions determine outcomes, making them more likely to resist external pressure. In contrast, those with an external LoC attribute outcomes to luck, fate, or powerful others and are thus more susceptible to conformity (Findley and Cooper, 1983; Lefcourt, 2014; Carstensen, 2024; Gebresilase et al., 2025). Empirical research consistently shows that externally oriented individuals are more easily persuaded and influenced by peers than those with an internal orientation (Shute, 1975; Rinn et al., 2014; Quick and Considine, 2008). These patterns are supported by social learning theory (Rotter, 1954) and attribution theory (Weiner, 1974; Weiner, 1985), which emphasize how beliefs about personal control shape behavioral responses.

Assertiveness also plays a central role in determining whether individuals conform or resist social influence. Assertiveness refers to the ability to express one’s feelings, defend one’s rights, and decline unreasonable requests while maintaining respect for others (Alberti and Emmons, 2008). Assertive individuals are more capable of resisting undue pressure, rejecting arbitrary authority, and maintaining independence from irrational group demands. Interventions that strengthen assertiveness have been shown to enhance self-esteem and psychological well-being, equipping individuals with greater capacity to withstand group influence (Golshiri et al., 2023). Internal LoC is consistently associated with greater assertiveness, which partly explains the lower susceptibility to conformity among individuals high in internal control (Sheinov, 2020). Conversely, low assertiveness often linked to shyness, low self-esteem, and dependence is associated with heightened vulnerability to social influence, particularly among those with an external LoC (Okechi et al., 2013; Nevid and Rathus, 2007). Assertiveness therefore represents a meaningful psychological pathway through which locus of control may shape conformity.

Although earlier studies have examined the relationships among locus of control, assertiveness, and conformity (e.g., Williams and Warchal, 1981; Avtgis, 1998), these investigations were largely correlational, conducted several decades ago, and focused predominantly on Western adult or college samples. They did not consider assertiveness as a mediating mechanism nor examine these processes in contemporary adolescent contexts where peer dynamics, cultural influences, and social pressures have evolved considerably. The present study extends this literature by testing an integrated mediation model in a culturally distinct adolescent population, applying modern measurement approaches including confirmatory factor analysis (CFA) and using validated scales suited for contemporary research. This framework allows for a more nuanced understanding of how locus of control influences conformity through assertiveness.

Taken together, these perspectives highlight the need to examine how personal dispositions and social-contextual factors jointly shape conformity in adolescents. The present study addresses this gap by testing a mediation model in which assertiveness mediates the relationship between locus of control and conformity among Ethiopian high school adolescents. By doing so, this research contributes theoretically and practically to understanding conformity within a non-Western adolescent population and offers insights for developing interventions that strengthen personal agency and reduce maladaptive conformity.

## Locus of control and conformity

Previous studies examining the relationship between locus of control and conformity have produced mixed findings, with some reporting significant associations while others found little to no relationship. For instance, Maadal (2020) reported no significant link between locus of control and conformity, though his findings indicated that males tended to conform more readily than females. In contrast, Oliner and Oliner (1998) conducted a historical study involving non-Jewish survivors of World War II, comparing those who had resisted Nazi orders and protected Jewish people with those who had complied. Their results revealed that the 406 “rescuers” who resisted orders were more likely to demonstrate a high internal locus of control than the 126 individuals who simply followed orders. These findings suggest that individuals with a strong internal locus of control may be less inclined to submit to authority. However, the authors also noted that multiple factors, beyond locus of control, likely influenced decisions during such extreme circumstances.

Additional research supports the notion that individuals with an internal locus of control are generally less susceptible to conformity pressures. For example, Spector (1983), using Rotter’s locus of control scale with a sample of 157 students, found that participants with a high internal locus of control were less likely to conform than those with a high external locus of control, but this effect was observed only in situations involving normative social influence where the motivation to conform is rooted in a desire for social acceptance. No significant differences emerged between internal and external groups under informational social influence, where the drive to conform stems from a need for accuracy. This pattern indicates that normative social influence plays a stronger role than informational influence in shaping the relationship between locus of control and conformity (Avtgis, 1998).

## Theoretical framework

The present study advances a focused process model informed by Social Learning Theory (Rotter, 1966) and Attribution Theory (Weiner, 1985). Together, these theories explain how adolescents’ beliefs about personal control shape their behavioral responses within social contexts. In this model, locus of control (LoC) functions as a distal cognitive determinant, assertiveness represents a behavioral self-regulation mechanism, and conformity emerges as a social influence outcome. Although these constructs are theoretically linked in prior work, their integrated operation has seldom been tested empirically particularly in non-Western settings such as Ethiopia.

### Locus of control and social learning theory

According to Social Learning Theory, behavior is shaped by expectancy and the value individuals assign to reinforcement. LoC reflects a generalized expectancy: adolescents with an internal LoC believe outcomes stem from their own actions, whereas those with an external LoC attribute outcomes to external agents such as fate, luck, or powerful others (Rotter, 1966). Within the domain of social influence, these expectancies translate into meaningful behavioral differences. Internal individuals tend to rely on personal judgment and show greater independence, whereas externals are more susceptible to peer norms and social expectations (Findley and Cooper, 1983; Lefcourt, 2014). Thus, Social Learning Theory provides the foundation for understanding how generalized expectancies develop and why they predispose adolescents toward broader behavioral patterns such as independence or conformity. In this study, internal and external LoC are conceptualized as related but distinct dimensions, rather than opposite poles of a single continuum. Although Rotter’s (1966) original formulation treated LoC as bipolar, subsequent theoretical developments (Levenson, 1974; Lefcourt, 2014) show that individuals may simultaneously hold beliefs in personal agency (internal control) and beliefs regarding the influence of external forces such as peers, authority figures, or chance. This multidimensional conceptualization is particularly salient in collectivistic contexts such as Ethiopia, where adolescents may value personal responsibility while also recognizing strong social expectations and hierarchical norms.

Consistent with this framework, internal and external LoC were treated as separate predictors within the same analysis. This approach enables the estimation of their unique effects on assertiveness and conformity while controlling for shared variance. Modeling them independently avoids assuming that one dimension necessarily increases as the other decreases and allows for the detection of culturally meaningful differences in their behavioral pathways. This analytic strategy aligns with evidence that internality and externality can independently predict psychosocial outcomes (Findley and Cooper, 1983; Levenson, 1974).

### Attribution theory and the cognitive mechanism

Attribution Theory (Weiner, 1985) elaborates on the cognitive processes that underlie these expectancies. Whereas Social Learning Theory clarifies *what* expectancies individuals hold, Attribution Theory clarifies *why* they interpret events in particular ways. Internal attributions such as attributing outcomes to effort or ability enhance perceived agency, promote self-regulation, and reduce reliance on external validation. In contrast, external attributions linked to luck, social pressure, or task difficulty encourage dependency on outside forces and increase sensitivity to social influence. This attributional reasoning provides the psychological basis for understanding why external LoC leads to higher conformity: adolescents who frequently attribute outcomes to external forces view personal assertion as ineffective, making deference to the group more likely.

### Assertiveness as a mediator

Assertiveness is positioned as the behavioral mechanism that connects LoC to conformity. From a social learning perspective, adolescents with internal LoC are more inclined to develop assertive behaviors because they believe personal action can produce meaningful outcomes. Conversely, those with external LoC perceive limited personal control and thus see less value in assertiveness, increasing their tendency toward compliance. At the same time, assertiveness embodies the cognitive evaluations described in Attribution Theory. Believing that one’s input matters (internal attributions) strengthens assertive responses, while external attributions diminish them. Empirically, assertiveness has been shown to reduce susceptibility to undue peer influence, whereas low assertiveness heightens conformity (Alberti and Emmons, 2017; Okechi et al., 2013).

### Current study and hypothesis

This integrated model is especially relevant within the Ethiopian cultural context an emerging economy characterized by collectivist norms, interdependent social relationships, and strong expectations for respect and hierarchical deference (Habtamu, 2002). In such environments, conformity is not only a personal disposition but also a socially reinforced behavioral expectation. These cultural dynamics intensify the theoretical relationships under examination: external LoC may be more common or socially encouraged, and the cost of deviating from group norms may be higher. Exploring this pathway within Ethiopia therefore provides an opportunity to assess whether the sequence from LoC to assertiveness to conformity is amplified, altered, or uniquely shaped by cultural values that prioritize social cohesion. This contextual grounding strengthens the study’s contribution by evaluating the cross-cultural validity and potential boundary conditions of the proposed framework.

### Research hypotheses

Based on theoretical frameworks and prior empirical evidence, the study proposes the following hypotheses:

*H1:* Locus of control significantly predicts conformity among students.

H1a, students with an external locus of control are expected to report higher conformity, H1b, internal locus of control are expected to report lower conformity.

**Figure 1:**
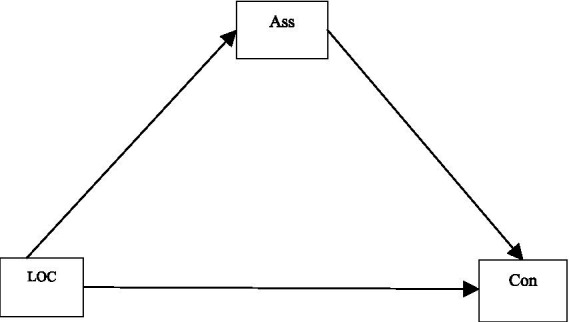
The study model LOC refers locus of control, Ass refers assertiveness, Con refers conformity.

*H2:* Assertiveness mediates the relationship between locus of control and conformity. Students with an internal locus of control are expected to demonstrate higher assertiveness, which in turn predicts lower conformity (see Figure 1).

## Methods

This study utilized a cross-sectional research design to examine the mediating role of assertiveness in the relationship between locus of control and adolescent conformity. It is important to acknowledge that while mediation is traditionally tested using longitudinal designs to establish causal pathways (Maxwell and Cole, 2007). Mediation analysis using cross-sectional data was conducted in this study to explore potential indirect relationships. The interpretation of mediation results is therefore exploratory and should be viewed with caution, recognizing the inherent limitations of causal inference in cross-sectional designs (Rucker et al., 2011). Data were collected through self-report questionnaires completed by adolescents. To analyze the hypothesized mediation models, Hayes’ PROCESS macro (Version 4.0) for SPSS was employed (Hayes, 2018).

### Participants

Data for this study were collected through a cross-sectional survey conducted among adolescents in Southern Ethiopia. Participants were recruited from four secondary schools, three public and one private located in the capital city of Southern Ethiopia, approximately 385 km from Addis Ababa. These schools were purposefully selected to represent variation in school type and educational context within the city. Although all four adhere to the national curriculum, they differ modestly in resources, class size, and student–teacher ratios, which are common distinctions between public and private schools in Ethiopia. To minimize potential school-level bias, the sample size drawn from each school was determined proportionally to the student population, and school identifiers were later included as control variables in the analysis.

Following school selection, stratified sampling based on gender and grade level (grades 9–12) was used to ensure balanced representation. Within each stratum, simple random sampling was applied to select participants. A total of 516 adolescents completed the survey. The mean age of participants was 14.77 years (SD = 0.71). Of the total sample, 271 (52.52%) were male and 245 (47.48%) were female.

### Procedure

The survey was administered in a classroom-based setting during regular school hours. After obtaining official permission from each participating school, the research team visited selected classrooms and provided students with a clear explanation of the study’s purpose and procedures. Participation was voluntary, and students were informed that they could decline or withdraw at any time without penalty. No identifying information was collected, and students were assured that their responses would remain completely anonymous. The questionnaires were completed using paper-and-pencil forms. To protect privacy, students were seated apart, and teachers were not present during data collection. A trained research assistant supervised the administration of the survey in each classroom, provided clarification when needed, and ensured that students completed the questionnaire independently. Because Ethiopia is a multilingual context, the survey instruments were translated into the nation’s official working language prior to data collection. Two experienced language instructors both native speakers and faculty members in the Department of English as a Foreign Language conducted the translation. The translation and back-translation processes followed Brislin and Berry’s (1986) guidelines, ensuring linguistic accuracy and conceptual equivalence between the English and translated versions. Discrepancies were reviewed and resolved by the translation team. Ethical approval for the study was obtained from the Wolaita Sodo University Research Committee. In addition, formal authorization was secured from the participating schools. Students received clear instructions on how to complete the questionnaire, and data collection proceeded only after confirming their informed assent.

## Measures

### Locus of control

Internal and external locus of control were measured using Rotter’s (1966) 23-item scale. Sample items include statements such as “I am usually able to protect my personal interests” (internal locus of control) and “When I get what I want, it’s usually because I’m lucky” (external locus of control). Participants responded on a five-point Likert scale ranging from strongly disagree to strongly agree. The scale demonstrated strong reliability, with a Cronbach’s alpha of 0.88. A confirmatory factor analysis (CFA) was also conducted to assess the fit of the translated scale to the data. The CFA results showed good model fit, with χ^2^/df = 2.62, CFI = 0.96, TLI = 0.95, SRMR = 0.03, and RMSEA = 0.053.

### Conformity

Conformity was measured using an 11-item inventory originally developed by Mehrabian and Stefl (1995). Sample items include statements such as, “I often rely on, and act upon, the advice of others.” Participants rated each item on a four-point scale from “rarely” to “always.” Item scores were averaged, with higher scores reflecting greater conformity. Reliability analysis showed strong internal consistency, with a Cronbach’s alpha of 0.82. A confirmatory factor analysis (CFA) was also performed to evaluate how well the translated scale fit the data. The CFA results indicated an excellent model fit, demonstrated by χ^2^/df = 2.44, CFI = 0.97, TLI = 0.96, SRMR = 0.03, and RMSEA = 0.04.

### Assertiveness

Assertiveness was measured using a 20-item inventory developed by Lima et al. (2024). An example item is, “I am able to express my opinions even when they differ from those of my peers.” Participants rated each item on a four-point scale ranging from “rarely” to “always.” Item scores were averaged, with higher values indicating stronger assertiveness. The scale demonstrated excellent reliability, with a Cronbach’s alpha of 0.89. A confirmatory factor analysis (CFA) was also performed to examine the fit of the translated assertiveness measure. The CFA results showed that the model fit the data well, with χ^2^/df = 3.13, CFI = 0.96, TLI = 0.94, SRMR = 0.02, and RMSEA = 0.04.

### Data analysis

Descriptive statistics and bivariate Pearson correlation coefficients were first computed to examine initial associations among the study variables. Mediation analyses were then conducted to test whether assertiveness mediates the relationship between locus of control and adolescent conformity. All analyses were performed using Hayes’ PROCESS macro (version 4.0) in SPSS version 25.0. PROCESS estimates the indirect effect using the product-of-coefficients approach, where the indirect effect is calculated as the product of the effect of locus of control on assertiveness (path a) and the effect of assertiveness on conformity (path b), controlling for locus of control. To obtain robust estimates, 5,000 bootstrap samples with bias-corrected 95% confidence intervals (CIs) were generated. An indirect effect is considered significant when the CI does not include zero.

Because PROCESS calculates mediation using unstandardized regression coefficients, all variables were analyzed in their original scales to ensure correct estimation and interpretation of indirect effects. School identifiers and demographic covariates were included to adjust for potential clustering and confounding.

## Results

### Preliminary analysis

Table 1 presents the means, standard deviations, and Pearson correlation coefficients for all major study variables. The correlation analysis provided the foundational bivariate relationships for the study. Notably, and consistent with our conceptualization of them as related but distinct constructs, internal and external locus of control were positively correlated (*r* = 0.21, *p* < 0.05). This initial relationship supports the decision to model them simultaneously in subsequent analyses to isolate their unique effects. As hypothesized, internal locus of control demonstrated a strong negative association with adolescent conformity (*r* = −0.56, *p* < 0.01) and a significant positive correlation with assertiveness (*r* = 0.42, *p* < 0.01). External locus of control, meanwhile, was positively correlated with conformity (*r* = 0.39, *p* < 0.01) and negatively correlated with assertiveness (*r* = −0.33, *p* < 0.01). Assertiveness exhibited a meaningful negative correlation with conformity (*r* = −0.38, *p* < 0.01). This pattern of bivariate correlations provides initial support for the proposed mediation model, suggesting that internal and external LoC have opposing relationships with assertiveness and conformity. However, these zero-order correlations do not account for the shared variance between the LoC constructs. The subsequent multiple parallel mediation analysis was therefore conducted to estimate the unique direct and indirect pathways for each dimension.

**Table 1:** Descriptive and bivariate correlations results of study variables.

Variable	1	2	3	4
ILOC	–			
ELOC	0.21*	–		
CON	−0.56**	0.39**	–	
ASS	0.42**	−0.33**	−0.38**	–
M	1.32	1.22	0.53	1.05
SD	0.36	0.21	0.02	0.15

ILOC, internal locus of control; ELOC, external locus of control; CON, conformity; ASS, assertiveness; **p < 0.01, *p < 0.05.

### Construct validity and reliability

In addition to the confirmatory factor analysis (CFA) results reported in the Measures section which demonstrated good model fit for each construct and provided initial evidence of construct validity we further examined the psychometric properties of the scales using Average Variance Extracted (AVE), Composite Reliability (CR), and the Heterotrait–Monotrait ratio (HTMT). All constructs exhibited sufficient internal consistency (CR ≥ 0.70) and acceptable convergent validity (AVE ≥ 0.50), as shown in Table 2. Furthermore, all construct pairs had HTMT values below 0.85, indicating discriminant validity. Taken together, the CFA evidence and these reliability and validity indices confirm that the study’s measures are psychometrically sound and appropriate for subsequent mediation analyses.

**Table 2:** Construct validity and reliability.

Construct	AVE	CR	HTMT (vs other constructs)
Locus of control	0.62	0.89	0.53 (vs ASS)/0.44 (vs Con)
Assertiveness	0.71	0.90	0.42 (vs LOC)/0.52 (vs Con)
Conformity	0.66	0.93	0.47 (vs LOC)/0.56 (vs ASS)

AVE, Average Variance Extracted; CR, Composite Reliability; HTMT, Heterotrait-Monotrait ratio.

### Mediation analysis

This study tested a multiple parallel mediation model to examine whether assertiveness mediates the relationship between locus of control (conceptualized as two related but distinct constructs) and adolescent conformity. Crucially, both internal and external locus of control were included as simultaneous predictors in the model to estimate their unique direct and indirect effects, while controlling for their shared variance. The overall model revealed a significant pattern of effects. After controlling for external locus of control, internal locus of control remained a significant negative predictor of conformity. The unique indirect effect of internal locus of control through assertiveness was significant (indirect = −0.18, 95% CI [−0.261, −0.104]). This indicates that even when accounting for a person’s level of external LoC, a stronger belief in personal control still reduces conformity by fostering greater assertiveness.

**Figure 2:**
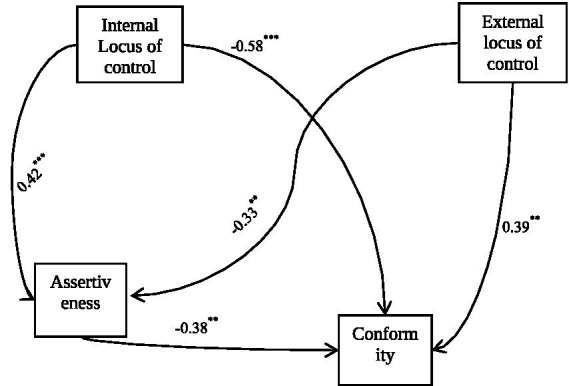
Mediation summary.

Simultaneously, after controlling for internal locus of control, external locus of control maintained a significant positive association with conformity. Its unique indirect effect via assertiveness was also significant (indirect = 0.10, 95% bootstrap CI [0.183, 0.211]). This shows that, independent of one’s sense of personal control, a stronger belief in external forces increases conformity partly by diminishing assertiveness. Overall, these findings from the simultaneous model provide robust evidence that assertiveness serves as a key psychological mechanism. Internal and external LoC exert distinct, independent influences on adolescent conformity through their opposing relationships with assertiveness. Internal LoC reduces conformity both directly and indirectly by boosting assertiveness, whereas external LoC increases conformity both directly and indirectly by reducing assertiveness. The path coefficients for the model are presented in Figure 2 and specific indirect effects were shown in Table 3.

**Table 3:** Specific indirect effects of locus of control on conformity through assertiveness.

Model path ways	Effect	Boot SE	Bias-corrected 95% CI	
			Lower	Upper
ILOC → ASS → Conformity	−0.18	0.04	−0.261	−0.174
ELOC → ASS → Conformity	0.10	0.02	0.183	0.211

## Discussion

Locus of control and assertiveness are essential psychological constructs that influence how adolescents navigate peer pressure and make autonomous decisions. Conformity during adolescence has long been recognized as a central developmental challenge because it increases vulnerability to peer influence, harmful decision-making, and maladaptive behavior patterns (Steinberg and Monahan, 2007). High susceptibility to conformity can shape long-term developmental trajectories, affecting social competence, academic choices, and psychological well-being. This is especially relevant in the context of the present study, conducted within a collectivist cultural setting where social harmony and alignment with group expectations are highly valued (Triandis, 1995). In such contexts, adolescents experience substantial social pressure to behave consistently with family, peer, and community norms. Those with an external locus of control may be particularly susceptible, as they tend to view outcomes as governed by fate, authority figures, or situational forces. Likewise, adolescents with limited assertiveness may find it challenging to resist normative pressures due to fears of rejection or disapproval.

The findings of this study align with theoretical expectations and prior empirical work. Internal locus of control was negatively associated with conformity, whereas external locus of control was positively linked to conformity. This suggests that adolescents who believe their outcomes arise from personal effort and ability are less likely to yield to social pressure, while those who attribute outcomes to external factors are more easily influenced. Consistent with this pattern, assertiveness emerged as a significant protective factor, with higher assertiveness predicting lower conformity. Assertive adolescents appear more capable of expressing their viewpoints, maintaining personal boundaries, and resisting coercive peer dynamics.

These results are consistent with findings from both Western and non-Western contexts. Prior studies have shown that internal locus of control is associated with reduced susceptibility to negative peer influence and more adaptive behavioral choices (Chubb et al., 1997; Galvin et al., 2018). Assertiveness has similarly been linked to resilience against risky behaviors and improved negotiation of social pressures (Zimmer-Gembeck and Collins, 2003). More recent cross-cultural research suggests that while conformity pressures may be heightened in collectivist societies, the protective effects of internal locus of control and assertiveness remain robust (Kim, 2020; Kumar and Hamid, 2021). These findings underscore the potential value of strengthening internal locus of control and assertiveness in adolescents, particularly in cultural settings where conformity is deeply embedded in social expectations.

### Theoretical contributions of the integrated mediation model

The present study makes two key theoretical contributions to the locus of control literature. First, by modeling internal and external locus of control as simultaneous predictors in a multiple parallel mediation model, we demonstrate that these dimensions exert independent and opposing effects on adolescent conformity through assertiveness. This advances beyond earlier work (e.g., Rotter, 1966) that treated locus of control as a bipolar continuum, as well as studies that examined each dimension in isolation. Our findings support the multidimensional perspective (Levenson, 1974; Lefcourt, 2014) by showing that individuals can simultaneously hold beliefs about personal agency and external forces, and that both beliefs uniquely shape social behavior. Had we analyzed each dimension separately, the unique contribution of each controlling for the other would have remained obscured.

Second, we identify assertiveness as a specific mediating mechanism linking control beliefs to conformity. While previous research has established direct relationships between locus of control and conformity, few studies have explored the psychological processes underlying these associations. By demonstrating that assertiveness partially mediates both pathways, our integrated model provides a more complete explanation of how control beliefs translate into social compliance or resistance during adolescence. This advances theoretical understanding beyond simple bivariate associations toward a more nuanced process-oriented model.

### Assertiveness as a mediator

A central objective of this study was to examine whether assertiveness mediates the association between locus of control and conformity. The mediation analysis supported this hypothesis. The indirect effect of internal locus of control on conformity through assertiveness was statistically significant (*b* = 0.20, *p* < 0.001), indicating partial mediation. This means that adolescents with a stronger internal locus of control are less likely to conform in part because they tend to be more assertive. Importantly, assertiveness significantly reduced the likelihood of conformity even among adolescents who scored higher on external locus of control. In such cases, assertiveness appears to mitigate the impact of external reliance by enhancing adolescents’ capacity to express their views, assert personal preferences, and resist normative pressures. This aligns with previous research demonstrating that assertiveness-building interventions help adolescents make autonomous decisions and resist maladaptive peer influence (Eslami et al., 2016). Similarly, Helweg-Larsen and Collins (1997) found that assertive individuals are more likely to reject social pressure to engage in health-risk behaviors often rooted in conformity needs during adolescence. From a developmental lens, adolescence is characterized by heightened sensitivity to peer acceptance (Laursen and Veenstra, 2021). Assertiveness functions as a critical social–emotional skill that helps adolescents communicate confidently, set boundaries, and counteract the urge for blind conformity. As such, assertiveness strengthens the influence of an internal locus of control while buffering the risks associated with external control orientations. Taken together, these findings indicate that locus of control and assertiveness operate interactively to shape conformity behavior. Locus of control provides adolescents with a general orientation toward autonomy or external dependence, whereas assertiveness acts as a practical skill that enables them to express autonomy in real-life social situations.

### Cultural implications of the Ethiopian context

The Ethiopian cultural context is an important factor that adds depth to what is observed and must be made specific. Ethiopian culture is inclined toward collectivist behaviors in which family considerations, peer group influences, and obedience to authority are highly prized (Spinelli et al., 2023). In such an environment, conformity is not merely an effect of peer pressures; it can be an expression of an idealized goal of interdependence. The finding that an external locus of control can facilitate conformity by inhibiting assertiveness could be especially pertinent in the present context: youths who believe that powerful others or fate control outcomes could be prioritizing group cohesion over personal assertiveness in line with local cultural priorities. The need to conform is not always driven by negative forces; it can also represent an adaptive fit with prosocial community values.

On the flip side, internal locus of control seems to protect against conformity by enhancing assertiveness, illustrating that believing in internal control can go along with collectivist beliefs. This is an interesting finding that challenges the simplistic individualism–collectivism dichotomy. Ethiopian adolescents that combine internal control with assertiveness might actually be able to cope with conformity pressures in an effective way: they can stay authentic to themselves while also respecting group pressures. This has implications for how to develop programs in such settings. Programs that aim to prevent negative conformity behaviors, like risky peer behaviors, do not need to eliminate conformity entirely. Rather, they need to develop teens’ ability to make nuanced judgments about when to stand up for themselves and when to conform to group behaviors in ways that fit the culture and serve them personally. Future research could investigate if this is found in other collectivist settings and how modernization/globalization is impacting these processes for adolescent.

## Conclusion

This study demonstrates that adolescent conformity is significantly influenced by locus of control and assertiveness, particularly within collectivist settings that emphasize social harmony. The findings reveal that individuals with an external locus of control exhibited a greater susceptibility to peer influence, whereas those with an internal locus were more likely to demonstrate independent judgment.

A key finding is the mediating role of assertiveness in the relationship between locus of control and conformity. This indicates that an internal locus of control fosters resistance to conformity not only through a belief in personal agency but also by promoting assertive behavior. Furthermore, assertiveness itself functions as a critical protective factor, mitigating conformist tendencies even among adolescents who lean toward an external locus of control.

These results underscore the practical importance of implementing educational and psychosocial programs designed to strengthen internal control beliefs and assertive communication skills. Empowering adolescents with these competencies can support their ability to make autonomous, healthy choices in the face of social pressures. For future research, longitudinal studies are recommended to assess the lasting impact of assertiveness training, and cross-cultural comparisons would be valuable to explore the universality of these dynamics.

### Implications

The findings carry significant theoretical and practical implications. Theoretically, the study extends understanding of how interpersonal skills such as assertiveness and personal dispositions like locus of control interact to shape adolescent behavior within collectivist cultural contexts. Practically, it provides evidence that the protective role of assertiveness remains consistent across varying levels of locus of control, reinforcing its value as a vital developmental competency.

Practically, the findings emphasize the importance of designing and implementing educational and psychosocial interventions that strengthen assertiveness and emotional regulation. For teachers and policymakers, this involves fostering adolescents’ confidence, promoting effective communication, and equipping them to resist harmful peer pressure by embedding assertiveness training within school curricula and extracurricular programs. Such interventions can empower young people to make healthier and more independent choices while still honoring group values and relationships in collectivist contexts where conformity is strongly reinforced. Parents and educators can also be encouraged to adopt autonomy-supportive practices that cultivate an internal locus of control and enhance resilience against conformity pressures.

### Limitations

Despite its valuable contributions, this study is not without limitations. First, the cross-sectional design limits the ability to draw causal conclusions about the relationships between locus of control, assertiveness, and conformity. Longitudinal studies would be beneficial to track how these variables interact over time during adolescence. Second, the reliance on self-report measures may introduce biases such as social desirability, particularly in collectivist contexts where conformity is culturally valued. Future research could incorporate multiple data sources, including peer, teacher, or parent reports, as well as behavioral assessments. Third, the study was conducted within a specific cultural setting, which may limit the generalization of the findings to other cultural contexts. Cross-cultural comparative studies could provide further insight into how cultural norms shape these relationships. Finally, while assertiveness emerged as a significant mediator, other potential mediators or moderators such as self-esteem, social support, or cultural identity were not examined and should be considered in future research.

## Data Availability

The raw data supporting the conclusions of this article will be made available by the authors, without undue reservation.
